# Rapid‐eye‐movement sleep‐predominant central sleep apnea relieved by positive airway pressure: a case report

**DOI:** 10.14814/phy2.13254

**Published:** 2017-05-08

**Authors:** Noah P. Jouett, Michael L. Smith, Donald E. Watenpaugh, Maryam Siddiqui, Maleeha Ahmad, Farrukh Siddiqui

**Affiliations:** ^1^ Institute for Cardiovascular and Metabolic DiseaseUniversity of North Texas Health Science CenterFort WorthTexas; ^2^ Sleep Consultants of TexasFort WorthTexas; ^3^ Department of Family MedicineUniversity of North Texas Health Science CenterFort WorthTexas

**Keywords:** Carbon dioxide, chemoreceptor, chemoreflex, oxygen, sleep apnea

## Abstract

Central Sleep Apnea (CSA) is characterized by intermittent apneas and hypopneas during sleep that result from absent central respiratory drive. CSA occurs almost exclusively during non‐rapid‐eye‐movement (NREM) sleep due to enhanced neuronal ventilatory drive during REM sleep that makes central apneas highly unlikely to form. A 45‐year‐old obese African American female presented with co‐existing Obstructive Sleep Apnea (OSA) and CSA, not in the form of mixed or complex sleep apnea. Peculiarly, her CSA occurred only during rapid‐eye‐movement (REM) sleep, which is exceedingly rare. The patient's CSA was resolved when appropriate positive airway pressure (PAP) was prescribed. Our patient remains stable and has reported significant benefit from PAP usage. We offer possible neuro‐physiological mechanisms herein, including enhanced loop gain and/or malfunction or malformation of the pre‐Botzinger nucleus or other neurological process, that could explain the unique findings of this case.

## Introduction

Central sleep apnea (CSA) is a relatively uncommon form of sleep‐disordered breathing (estimated to be less than 1% in the general population), and is characterized by reduced or absent respiratory effort in spite of the presence of hypoxic hypercapnia (Dempsey et al. 2010). The etiology of CSA is complex, and generates a long list of differential diagnoses including congestive heart failure and pulmonary arterial hypertension (Dempsey et al. 2010). Furthermore, CSA almost exclusively occurs during non‐rapid eye movement (NREM) sleep, and very rarely occurs during rapid‐eye movement (REM) sleep when usually chemosenstivity to CO_2_ is comparable to wakefulness, secondary to a noradrenergic dependent increase in neuronal ventilatory drive (Steffen et al. 2010; Yuceege et al. 2013). A patient that presented to our clinic was discovered to have co‐existing CSA and Obstructive Sleep Apnea (OSA), which is much more common and is characterized by an obstruction to ventilation in the presence of an intact ventilatory drive (Dempsey et al. 2010). The remarkable findings that characterize this case primarily involved prominent episodes of CSA occurring *only* during REM sleep, and the resolution of the central sleep‐disordered breathing with sufficient positive airway pressure. Details of this patient presentation, polysomnographic findings and discussion of the likely mechanisms that explain this unique case are described below.

## Case Presentation

### Patient presentation

A hypertensive, obese 45‐year‐old African American female presented with a history of several years of snoring, daytime sleepiness, and witnessed apneas. Sleep disordered breathing was suspected and a polysomnogram was obtained. Physical examination was unremarkable. The patient's vital signs included resting blood pressure of 135/88 mmHg, heart rate of 86 beats/min, respiratory rate of 16 breaths/min, oral temperature of 98.7°F, oxygen saturation of 96% and a BMI of 48 kg/m^2^. In addition, pulmonary function testing was performed and revealed an FEV_1_/FVC ratio = 75% of predicted and FEV_1_ = 46% of predicted. Finally, the patient reported a high level of pain (8/10), and the only prescription medication was Lisinopril (10 mg). The patient did not report any narcotic use, although a urine drug screen (UDS) was not obtained.

### Polysomnography findings

**Figure 1:**
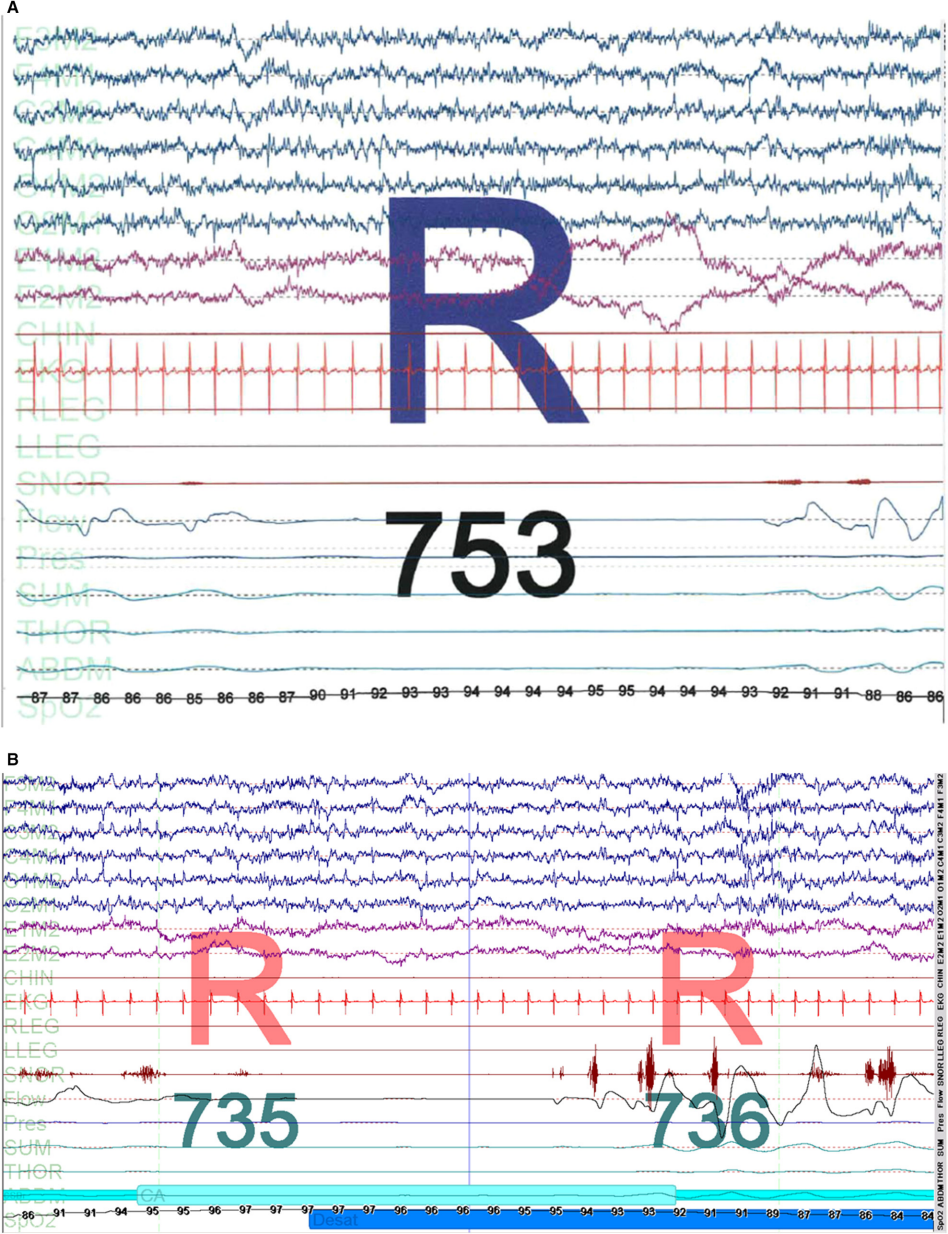
A–B: Baseline polysomngraphic tracing from our patient. Note the frank central events that occur exclusively during both phasic (A) and tonic (B) REM sleep and that do not have an obstructive component, hence making this inconsistent with mixed sleep apnea. Furthermore, the CSA observed in this study was not in the setting of PAP treatment and hence is also inconsistent with complex or treatment‐emergent sleep apnea (CompSAS).

The baseline polysomnogram showed severe sleep apnea, worse in REM sleep, both in the form of obstructive sleep apnea and central sleep apnea. Only lateral sleep was recorded. Peculiarly, frank central apneic events were seen only in REM sleep, and there was no evidence of Cheyne‐Stokes respiration. These central events occurred mostly during phasic REM, while a minority of events occurred during tonic REM. Figures 1A–B show sample tracings of the patient's polysomnogram that illustrate frequent CSA events occurring during REM sleep with each episode resulting in O_2_ desaturations to 80–85%. Polysomnography data are provided in Table 1. The patient then underwent a CPAP titration study and CPAP settings of 5–12 cm of water with c‐flex 3 were tested. Lateral sleep was observed throughout the entire recording. CPAP settings as low as 8 cm of water were effective in eliminating obstructive events in NREM sleep. However, in REM sleep, central apneas with periodic breathing patterns were observed at CPAP 9–10 cm of water with oxygen saturation dropping to 82%. At CPAP of 11 cm of water, there was one central apnea that produced no desaturation. At CPAP of 12 cm of water, there was complete resolution of the patient's sleep apnea, including both obstructive and central forms.

**Table 1:** Polysomnography data from the patient

	Initial polysomnography	Titration study^a^	At therapeutic pressure^b^
Time in Bed (min)	355	360	84.5
Total Sleep Time (min)	277.5	253	83
Stage N1 (%/min)	3.6/10	4/10	35/29
Stage N2 (%/minutes)	29.2/81	25.1/64	15/12
Stage N3 (%/minutes)	49.5/137	43.3/110	~1/<1
REM (%/minutes)	17.7/49	27.7/70	50/42
Obstructive Apneas	23	1	0
Hypopneas	96	30	4
Central apneas	39	14	1^c^
REM central apneas	39	14	1^c^
Mixed apneas	7	0	0
Time below 90% O_2_ saturation (%)	22.9	4.7	0

a Entire titration study including sub‐therapeutic pressures. Titration study was performed approximately 2 weeks after initial polysomnography.

b Portion of the titration study including 11‐12 cm H_2_O pressure.

c This central apnea did not cause any desaturation.

## Discussion

This case presents a unique phenomenon due to (1) the occurrence of CSA occurring during REM sleep and (2) the observation that sufficient positive airway pressure that eliminated the REM predominant CSA.

### Pathophysiology of central sleep apnea and its relationship with obstructive sleep apnea

CSA is marked by reduced or absent respiratory effort, indicating a lack of central ventilatory motor neuron discharge. The possible etiologies of CSA include pulmonary arterial hypertension, heart failure and neuropathologies such as mass‐occupying lesions in the brain and spinal cord (Dempsey et al. 2010). The reduction in ponto‐medullary influence on respiratory motor control seen in CSA is largely due to alterations to the physiologic chemosensitivity to P_a_CO_2_, which is primarily sensed by the central hypothalamic chemoreceptors. Physiologically, when P_a_CO_2_ is reduced, ventilation is inhibited centrally, thus allowing P_a_CO_2_ levels to rise globally. Yet, during wakefulness, an *extreme* reduction in P_a_CO_2_ is required to inhibit ventilation to the point of apnea (Dempsey et al. 2010). The central control of respiratory motor output prevents apnea when P_a_CO_2_ is physiologically reduced (Dempsey et al. 2010). However, during non‐rapid eye movement (NREM) sleep, central respiratory control is diminished, and a resulting apneic threshold of P_a_CO_2_ emerges (Dempsey et al. 2010). During NREM sleep, when ventilatory fluctuations occur that decreases P_a_CO_2_, the P_a_CO_2_ can easily fall below the threshold and provoke a central apnea. This situation can occur in the setting of OSA, where a high loop gain (i.e., a high ventilatory response‐to‐stimulus ratio, often seen in OSA) causes an exaggerated hyperventilation that drives P_a_CO_2_ beneath the apneic threshold. In the setting of a high loop gain, minimal reductions in ventilation and accompanying changes in arterial blood gas would be sufficient to activate chemoreflex control of ventilation to produce a large increase in ventilation sufficient to drive P_a_CO_2_ beneath the apneic threshold. This phenomenon has been experimentally demonstrated during NREM sleep in humans (Dempsey et al. 2010) and dogs (Chow et al. 1994), where mechanically induced airway occlusions triggered central apnea following hyperpnea.

Normally, when CSA occurs during sleep, it is almost entirely a NREM phenomenon. Chemoreflex control of ventilation during REM sleep is similar to that of wakefulness, where the apneic threshold for CO_2_ lies well below steady‐state ventilation (i.e., eupnea), as aforementioned. Consequently, the REM predominance of our patient's CSA is particularly remarkable. Moreover, the observation that OSA often occurs with an elevated loop gain likely does not explain the central apneas and hypopneas observed in our patient, provided that the patient exhibited normal neurophysiology. Hence, we postulate that this patient exhibits abnormal neurophysiological control of breathing during sleep, and speculate on possible mechanisms below, after offering a brief overview of the neurophysiological control of breathing during sleep.

### Neurophysiological control of breathing during sleep

The pre‐Bötzinger (PreBöt) complex and the retrotrapezoid nucleus (RTN) have been shown to be the primary central nuclei involved in respiratory pattern generation and chemoreception during sleep (Li et al. 1999; McKay et al. 2005; Burke et al. 2014). During non‐rapid eye movement (NREM) sleep, the RTN is relatively predominant in controlling respiration, and inhibition of the RTN during NREM sleep results in substantial reductions in tidal volume and respiratory motor output (Burke et al. 2014). However, during REM sleep, the PreBöt normally assumes greater control of respiration. Animal studies have demonstrated that ablation of this population of neurons results in reductions of tidal volume and breathing frequency during REM sleep, and not during NREM sleep or wakefulness (McKay et al. 2005; McKay and Feldman 2008). Diminished or maladaptive function of the PreBöt has been implicated in the setting of OSA and CSA in animal models (McKay et al. 2005; McKay and Feldman 2008) and fMRI studies of human patients indicate decreased activity in regions that include the PreBöt (Macey et al. 2003, 2014).

### Possible mechanisms accounting for the pathophysiology observed in our patient

What then is inhibiting the central respiratory drive in our patient? As noted above, REM sleep is associated is marked suppression of physiological apneas. Hence, two components are needed: (1) the emergence of the apneic threshold during REM sleep and (2) a reduction in P_a_CO_2_ beneath the apneic threshold. The first component most likely results from a central disease process, such a neuroborreliosis (Steffen et al. 2010), mass‐occupying lesions infringing on the brainstem (Dempsey et al. 2010) or a structural or functional neurophysiologic malfunction of the central respiratory nuclei (such as those mentioned above), either of a congenital or pathologic origin (Javaheri and Dempsey 2013). This patient refused magnetic resonance imaging, which would perhaps uncover a neuropathology that may be present, particularly if a mass‐occupying lesion were suspected. Other MRI‐negative etiologies of our patient's neurophysiological aberrations include general causes for neurodegeneration, such as Multiple Systems Atrophy (MSA), Parkinson's Disease and/or Parkinson's dementia with Lewy Bodies (McKay et al. 2005). However, as previously mentioned, our patient did not present with any neurological symptoms at the time of presentation, albeit she may have presented with early symptoms in the form of sleep‐disordered breathing. Indeed, it is unclear what neurologic process would account for our patient's REM‐dependent respiratory depression, and previous investigations have similarly found little cause to this phenomenon, with patients presenting no neurological symptoms and demonstrating normal cranial MRI studies (Steffen et al. 2010; Yuceege et al. 2013). Alternatively, due our patient's BMI, these findings may be explained by either obesity‐hypoventilation syndrome, neuromuscular weakness, or both. The second component could result from the patient's underlying OSA, which, as aforementioned, could be accompanied by a high loop gain, which would in turn result in ventilatory overshoots that could drive P_a_CO_2_ beneath the apneic threshold. Intermittent hypoxia associated with obstructive sleep apnea can result in a high loop gain due to long‐term facilitation of respiratory controller neurons (Deacon and Catcheside 2014). Alternatively, systolic heart failure or pulmonary arterial hypertension could cause unstable ventilatory control consistent with periodic reductions in P_a_CO_2_ secondary to delayed circulation time. The patient denied pillow orthopnea, chest pain with exertion and dyspnea and furthermore pulmonary crackles and rales were not noted on physical exam, thus rendering the latter possibilities unlikely. Furthermore, this would likely manifest with a Cheyne‐Stokes respiratory pattern at rest which was not observed in this patient.

### Obstructive sleep apnea and co‐associated central sleep apnea

As in our patient, CSA frequently associates with OSA in the form of mixed sleep apnea. Mixed sleep apnea usually occurs where prolonged central events produce transient obstructive apneas (Khandoker et al. 2013). CSA also manifests itself in the context of OSA during Positive Airway Pressure (PAP) titration studies (and *not* during baseline polysomnography), which is termed treatment‐emergent or complex sleep apnea (CompSAS) and is thought to be related to Hering‐Breuer mechanisms via PAP‐mediated activation of pulmonary stretch receptors (DelRosso et al. 2014; Morgenthaler et al. 2014). However, our patient's CSA and co‐existing OSA was not in the form of mixed or CompSAS. As aforementioned, we speculate that the patient's OSA most likely caused the elevation in loop gain necessary to drive P_a_CO_2_ beneath the apneic threshold in the setting of decreased REM ventilatory drive and therefore produces central apneas.

## Treatment and follow‐up

After many years of struggling without an explanation of her symptoms, our patient was finally diagnosed with OSA and CSA. Continuous positive airway pressure (CPAP) was prescribed at 12 cm of water. The patient has done well on CPAP and has had improvement with daytime sleepiness. As of our most recent follow‐up appointment, the patient was functioning well with treatment. She reported her sleep to be minimally fragmented at night and she felt well rested upon awakening, thus mitigating her need for napping.

## Conclusion

This case represents a unique and rare finding in which CSA occurs predominantly during REM sleep and not during NREM sleep and is effectively reversed by CPAP. The mechanism remains uncertain, but the fact that CPAP eliminates the CSA points to a mechanism in which partial obstruction (during REM) contributes to a feedback that modulates central respiratory drive and culminates in CSA events, and thus can be relieved by sufficient positive airway pressure.

## Limitations

Our report is limited by our patient's refusal to obtain an MRI study. However, previous investigations of REM‐dependent CSA demonstrated normal MRI without any neurological impairments noted (Steffen et al. 2010; Yuceege et al. 2013). Furthermore, although animal studies have offered very definitive mechanistic explanations to neural control during REM sleep, evidence in human patients is lacking, which limits our discussion of the present case. Furthermore, although our patient did not exhibit signs or symptoms of heart failure, an echocardiogram was not obtained, which may have demonstrated some latent reduction in ejection fraction. Finally, end‐tidal or transcutaneous CO_2_ monitoring was not performed during our patient's polysomnogram, which may have offered some insight into a possible pulmonary etiology of our patient's condition.

## Ethics, consent and permissions

All reasonable efforts to obtain consent for publication from the patient's next of kin failed as they were untraceable. The authors have made every effort to ensure patient anonymity. There is no reason to believe that the patient would have objected to publication and it is not felt that anyone who knew the patient would be able to identify her from the published article. The review of the patient's records was approved by the University of North Texas Health Science Center Institutional Review Board (IRB # 2015‐192).

## Conflict of Interests

The authors declare that they have no competing interests.
